# Editorial: a new journal in bipolar disorders

**DOI:** 10.1186/2194-7511-1-1

**Published:** 2013-04-17

**Authors:** Michael Bauer

**Affiliations:** Department of Psychiatry and Psychotherapy, University Hospital Carl Gustav Carus, Technische Universität Dresden, Fetscherstr, 74, D-01307 Dresden, Germany

I am very pleased to welcome you to the launch of *International Journal of Bipolar Disorders* (*IJBD*), a new, peer-reviewed, open access, online journal. IJBD will publish contributions from the entire range of clinical, psychological and biological research in bipolar disorders. The need for a new journal in bipolar disorders is reflected by an increasing number of research activities around the globe in this disease area. With the help of a distinguished international editorial board of leading researchers from around the world, *IJBD* aims to help bridge the gap between research findings and clinical practice, as well as to facilitate the dissemination of new research data, with the ultimate goal of improving the care of patients with bipolar disorders. IJBD will be the official journal of the *European Network of Bipolar Research Expert Centres* (*ENBREC*), *the International Group for the Study of Lithium-Treated Patients* (*IGSLi;*http://www.igsli.org)*,* and the German Society for Bipolar Disorders: *Deutsche Gesellschaft für Bipolare Störungen* (http://www.dgbs.de).

Bipolar disorders cause severe suffering for patients and caregivers, and constitute a major health economic challenge for societies (Gustavsson et al. 2011). According to the most recent WHO global burden of disease study, bipolar disorders rank within the top 20 causes of disability among all medical conditions worldwide, and rank 6^th^ among the mental disorders (Vos et al. 2012). The life expectancy of patients with bipolar disorder is reduced by more than 10 years (Chang et al. 2011), likely due to medical comorbidity, high suicide rates and adverse lifestyles. Clearly, bipolar disorders deserve a higher priority in the research agenda to gain understanding of underlying pathogenic mechanisms, reduce the diagnostic delay, and improve treatment strategies. Although research activities have increased in many countries in the past decade, funding for research in bipolar disorder is still low compared with other severe mental and medical disorders.

My strong hope is that this new journal will facilitate the publication and global access to new research results, and foster the exchange of ideas among the multidisciplinary health professionals and researchers involved with bipolar disorders. We encourage clinicians and researchers to submit original research papers, short research communications, reviews, guidelines, exceptional clinical case reports and commentaries (letter to the editor) concerning bipolar disorders. IJBD will publish all content online, freely available to researchers worldwide, thereby ensuring maximum dissemination. Together with the Managing Editor Dr. Emanuel Severus, the editorial team and peer-review system, we will ensure that all submitted manuscripts are reviewed fairly and swiftly, and once accepted, published online rapidly. Please participate in *International Journal of Bipolar Disorders* as readers and authors. The journal’s new home is at http://www.journalbipolardisorders.com/.
